# Respiratory microbiota, host immunity, respiratory viral infections and malignant tumors

**DOI:** 10.3389/fmicb.2025.1626077

**Published:** 2025-07-10

**Authors:** Xinjie Jiang, Sujiang Zhang

**Affiliations:** ^1^College of Life Sciences and Technology, Tarim University, Alar, China; ^2^College of Life Science and Agriculture Forestry, Qiqihar University, Qiqihar, China; ^3^Key Laboratory of Tarim Animal Husbandry Science and Technology, College of Animal Science and Technology, Tarim University, Alar, China

**Keywords:** respiratory tract microbiota, viral infection, airway dysbiosis, immune, interaction

## Abstract

In recent years, the role of the respiratory tract microbiota in respiratory tract infections has attracted considerable attention. Respiratory microbiota have important effects on respiratory physiology, immune regulation, and the occurrence and development of various respiratory viral infectious diseases. The microbial composition in the different parts of the respiratory tract, such as the nose, oropharynx, and lower respiratory tract, varies. Under physiological conditions, the respiratory microbiota remains relatively stable; however, when this homeostatic balance is disrupted, respiratory microbiota imbalance occurs, increasing the risk of infection. An increasing number of studies have revealed the complex relationship between bacterial dysregulation and respiratory viral infections. Dysregulation of the respiratory tract microbiota plays an important role in both innate and adaptive immune responses. In this study, changes in respiratory microbes and their interactions with host immunity, respiratory viral infections and malignant tumors were reviewed. Future studies should further explore the interaction mechanism between respiratory microbiota and host immunity, develop new diagnostic and therapeutic strategies, and improve the current level of clinical treatment for respiratory diseases.

## 1 Introduction

Microbiota are found in all parts of the respiratory tract. Respiratory microbiota participate in the maturation of respiratory physiology and local immune regulation and are closely related to the occurrence and progression of various respiratory diseases. The human respiratory tract is anatomically divided into the upper and lower respiratory tracts. The upper respiratory tract includes the mouth, pharynx, and larynx, whereas the lower respiratory tract includes the trachea, bronchi, and lungs. Different anatomical parts of the respiratory tract have different microbial compositions (Zhou et al., 2024). When the microbiota structure deviates from the physiological state, microbiota dysregulation occurs, which manifests as a decrease in the abundance of probiotics and symbiotic bacteria and an increase in the abundance of potentially pathogenic bacteria (Natalini et al., 2023). Microbial dysregulation is closely related to the occurrence and development of various respiratory diseases, especially chronic airway diseases, such as chronic obstructive pulmonary disease and bronchiectasis (Chellappan et al., 2019). In recent years, increasing attention has been paid to respiratory tract infections, and outstanding progress has been made in research on the role of respiratory tract microbiota in respiratory tract infections. This paper reviews the recent findings on the relationship between the respiratory microbiota and host immunity, respiratory viral infections and malignant tumors, respectively.

## 2 Main distribution of respiratory microbiota

The adult respiratory microbiota has a different structure in different respiratory regions. The main microbes in the nasal cavity include *Staphylococcus*, *Propionibacterium*, *Corynebacterium*, *Moraxella*, and *Streptococcus*. The microbiota of the nasopharynx is similar to that of the nasal cavity, with dominant bacteria including *Moraxella*, *Staphylococcus*, *Corynebacterium*, and *Streptococcus*, and other major microbiota including *Dolosigranulum* and *Hemophilus*. Microbes in the oropharynx are very different from those in the nasal cavity and nasopharynx. In addition to *Streptococcus*, the genera *Prevotella*, *Veillonella*, *Rothia*, *Leptotrichia*, *Neisseria*, and *Fusobacterium* are present (Natalini et al., 2023; Man et al., 2017). The microbiota of the lower respiratory tract is relatively simple, with a low load, and is mainly composed of oral symbiotic bacteria, such as *Streptococcus, Veronella*, and *Prevotella*. The microbiota of the oropharynx can enter the lower respiratory tract through microinhalation or other means, thereby affecting respiratory health. The spread of oral microbiota to the lungs is heterogeneous, and enrichment in the lungs is associated with decreased lung function and increased lung proinflammatory cytokine levels (Zhang et al., 2022). The composition of the lung microbiota is tightly controlled by airway clearance mechanisms such as cough, mucociliary transport, and the innate immune system to prevent colonization and infection by pathogens. It is generally believed that nasal microbiota are the main source of healthy lung bacteria. The oral cavity is structurally adjacent to the respiratory tract and alveoli, and the oral microbiota directly affects the composition of the lung microbiota. Multiple studies have highlighted differences in microbial diversity, biomass, and community structure between the upper and lower respiratory tracts (Charlson et al., 2011; Morris et al., 2013; Li et al., 2024).

## 3 The effect on innate and adaptive immune responses

### 3.1 Innate immune response

**Figure 1:**
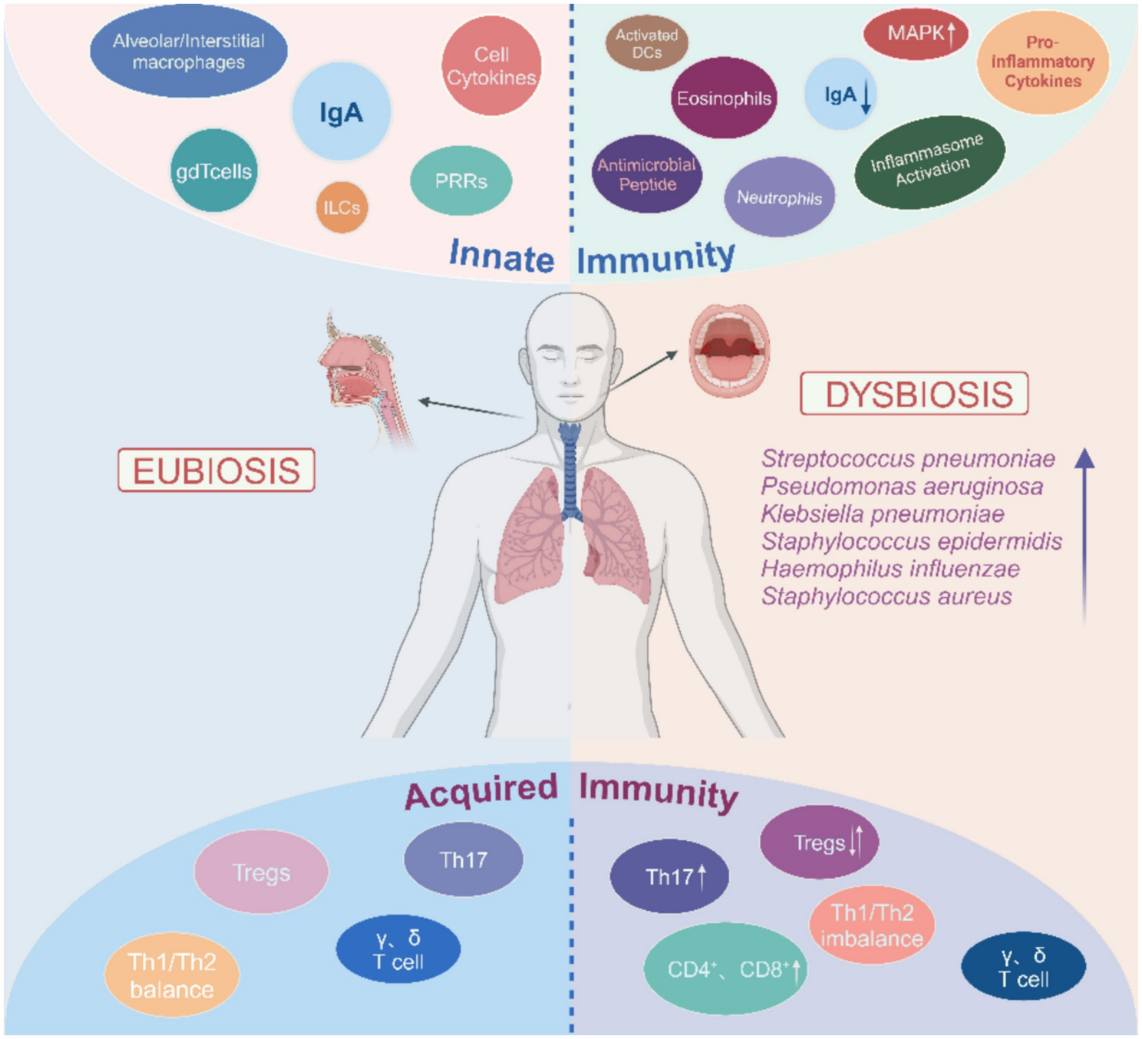
Overview of the respiratory tract microbiota dysbiosis and immunity. Respiratory tract microbiota dysbiosis leads to activation of immune cells. The immune cells then migrate into the tissue and produce pro-inflammatory cytokines, which ultimately contribute to the local inflam-matory response. In addition, changes in the cytokine environment promote pathological fibrotic remodeling, NETosis, and apoptosis. However, this also maintains the pulmonary immune balance through the differentiation of Tregs and Th17 cells. The arrows indicate changes in the relative abundance of immune cell subsets and bacterial species.

Host-microbial interactions affect different aspects of the immune system development, promoting immune maturation, immune tolerance, and immune response (Hu et al., 2020) (Figure 1). Lung epithelial cells, macrophages, and dendritic cells (DC) have different receptors to sense microbiota; these microbial pattern recognition receptors (PRR) include Toll-like receptors (TLRS) and NOD-like receptors (NLRs) (Uehara et al., 2007). Epithelial cells are involved in multiple mechanisms by which the microbiota in the lung interact and act as a permeable barrier, sensing microbiota and responding to their presence (Evans et al., 2010). By providing a strong barrier in the lower respiratory tract, the airway epithelium is the primary line of defense against potentially harmful environmental irritants. It is the first site to interact with inhaled compounds and is designed to promote the efficient removal of particles and microbiota by the mucous cilia (Leiva-Juarez et al., 2018). In chronic lung disease, the increased mucus produced by epithelial cells promotes bacterial growth and leads to low oxygen concentrations and areas of high temperature, which promote the selectivity and stability of specific bacteria (Invernizzi et al., 2020).

Human and mouse models have demonstrated the important role of innate immune responses in regulating microbiota variation, composition, and individual differences (Levy et al., 2015). In healthy mice, IL-1α is a key cytokine in anti-bacterial innate immune activity in the lungs and is negatively correlated with the diversity of bacterial communities and the presence of pathogenic bacteria. In addition, lung concentrations of this inflammatory cytokine is more closely associated with changes in the lung microbiota than with distal intestinal or oral concentrations (Dickson et al., 2018). Upper respiratory tract symbiotic bacteria defend against influenza virus infection in mice through polarization of M2 macrophages and secretion of anti-inflammatory mediators such as IL-10 and transforming growth factor-*β* (TGF-β) (Iwasaki and Medzhitov, 2015). Microbiota also produce metabolites associated with immune responses. For example, oral commensals generate short-chain fatty acids (SCFAs) that regulate the action of Treg or other immune cells and the expression of pro-inflammatory factors. These effects on immunity and inflammation can be direct or indirect, but all serve to maintain microbial homeostasis (Furusawa et al., 2013; Smith et al., 2013; Oh et al., 2019). Long-chain fatty acids (LCFA) also play a role in immunity but cannot be synthesized directly by mammals. Instead, microbial taxa (e.g., *Prevotella*, *Lactobacillus*, and *Alistipes*) in the gastrointestinal tract catabolize food to yield LCFAs (Zhao et al., 2018). In a cohort study, antibiotic treatment of pulmonary malignant transformation in cystic fibrosis led to significant LCFA upregulation, implying a link between LCFA and pulmonary inflammation (Hsu et al., 2015). Other major immunity-related metabolites include D-phenyllactic acid (D-PLA), produced by lactic acid bacteria and the most potent natural agonist of human hydroxycarboxylic acid receptor 3 (HCA 3). The latter is expressed in immune cells such as macrophages, neutrophils, monocytes, and adipose tissue (Zhou et al., 2023).

After transplanting respiratory microbiota from mice infected with chronic pneumonia into germ-free mice, lung inflammation increased the secretion of interleukin-17A (IL-17A) in germ free mice (Yadava et al., 2016). After nasal drops of bacteria rich liquid were administered to germ-free mice, the level of IL-17 increased. Lung inflammation of the model mice was significantly relieved after neutralizing IL-17, indicating that the increase in lung bacterial load mediates the increase in the level of the inflammatory factor IL-17 and aggravates lung inflammation (Mannion et al., 2023). Yang et al. (2019) analyzed and compared the bacterial community composition of normal lung tissue and pathological tissue of pulmonary fibrosis and found that outer membrane vesicles (OMVs) were secreted after the abundance of *Prevotella* and *Bacteroides* increased. OMVs carry lipopolysaccharides (LPS) and lipoproteins by acting on Toll-like receptors of alveolar macrophages. PRRs further activate the downstream myeloid differentiation factor 88 (MyD88) signaling pathway, thereby promoting the expression of interleukin-17B (IL-17B). IL-17B acts directly on the lung epithelial cells and causes pulmonary fibrosis by recruiting neutrophils and promoting the differentiation of Th17 cells (Tsai et al., 2013). Singanayagam *et al*.(Singanayagam et al., 2019) found that the disturbance of the pulmonary microbiota in patients with chronic obstructive pulmonary disease (COPD) was closely related to the decreased expression of cathelicidin, and increasing exogenous antimicrobial peptides inhibited the expansion of *Streptococcus pneumoniae* and reduced the total number of bacteria in the lung tissue. These results indicate that antimicrobial peptides may affect immune homeostasis by altering the structure of lung microbiota. In lung diseases, pathologic changes of lung structure and impaired mucous clearance mechanism may lead to microecological imbalance, and microbial imbalance may promote the occurrence and development of the disease by upregulating inflammatory signals such as NF-κB, Ras, IL-17, and PI3K, or inhibiting the production of TNF and IFN-*γ* in response to pathogens in the lower respiratory tract (Faniyi et al., 2022).

Certain microbiota, such as *S. pneumoniae*, promote a wide range of intrinsic responses in the respiratory tract, support the clearance of pathogens, and improve host survival during infection via IL-17 axis and granulocyte macrophage colony stimulating factor signaling (Brown et al., 2017). *S. pneumoniae* and *Hemophilus influenzae* can be activated by the mitogen-activated protein kinase (MAPK) signaling pathway, which causes inflammation in the lungs (Lynch, 2016). *Klebsiella pneumoniae* ST258 can change the metabolic response of the host, enhance glutamine metabolism and fatty acid *β* oxidation after glucose depletion, lead to an increase in lung active oxide content, recruit immunosuppressive cells, promote the secretion of anti-inflammatory factors, and prolong the survival time of pathogens (Dominguez-Andres et al., 2019).

When germ-free mice are inoculated with respiratory symbiotic microbes such as *Staphylococcus aureus* and *Staphylococcus epidermidis*, the granulocyte macrophage colony stimulating factor (GM-CSF) signaling pathway and NLRs are activated, and the NLRs signaling pathway improves resistance to infection (Brown et al., 2017). The lung microbiota of patients with lung cancer is significantly different from that of healthy people, and pathogens such as *Mycobacterium tuberculosis* can produce in-flammatory cytokines (such as IL-1β and IL-23) by activating the MyD88 signaling pathway, further stimulating lung γδT cells to produce IL-17 and other effector molecules to promote tumor cell proliferation (Mao et al., 2018; Jin et al., 2019).

### 3.2 Adaptive immune response

The pulmonary microbiota mainly induces the differentiation of Tregs and Th17 cells by producing PRRs to maintain the pulmonary immune balance. During the fetal period, the immune response is mainly dominated by Th2 cells because the immune system is immature. About two weeks after birth, the main members of the lung microbiota gradually change from *Proteobacteria* and *Firmicutes* to *Bacteroides*, promoting natural T cells from Th2 type immune response to Th1 type immune response, which can enhance resistance to asthma and allergic diseases (Wang et al., 2017). The enrichment of pulmonary oropharyngeal microbiota, such as *Veillonella* and *Prevotella*, has been associated with inflammatory phenotypes, including elevated Th17 lymphocyte levels, increased expression of inflammatory cytokines, and decreased expression of the inflammatory cytokine TLR4 in alveolar macrophages (Segal et al., 2016). In mice, neutrophil infiltration, high levels of IL-6 and TNF-*α*, and moderate levels of CD4^+^ T-cell-derived IFN-*γ* and IL-17 were associated with *Proteobacterium catarrhalis* infection (Alnahas et al., 2017). Inhalation of symbiotic bacteria induces a long-term immune response in healthy mice. This includes CD4^+^ and CD8^+^ T cell activation, Th17 and γδT cell recruitment, and other anti-regulatory immune responses, such as increases in Tregs and immune checkpoint inhibitor markers on T cells (Wu et al., 2021). Certain lung microbes, including *Staphylococcus*, produce short chain fatty acids that regulate changes in oral microbes (Remot et al., 2017). In the epithelial lining of patients in an immunocompromised condition, short chain fatty acids production was associated with elevated levels of Tregs induced by *M. tuberculosis* antigen (Remot et al., 2017; Segal et al., 2017).

The exact outcome of changes in microbial diversity in patients with lung cancer has not been elucidated; however, previous studies have shown that increased *α*-diversity is generally associated with improved survival and treatment outcomes in several cancers, such as cervical cancer, because α-diversity increases tumor invasion of CD4^+^ lymphocytes and expression of ki67^+^ and CD69^+^ (Sims et al., 2021). Lung tumor growth is associated with an increase in the number of bacteria in the airway and changes in bacterial composition; for example, a dysregulated protomicrobiome triggers MyD88 dependent IL-1 and IL-23 production, and induces the activation and proliferation of lung-resident Vγ6 ^+^ Vδ1^+^γδT cells (Jin et al., 2019).

The microbiota diversity in patients with pneumonia with acquired immune deficiency syndrome (AIDS) is higher than that in patients without AIDS (Twigg et al., 2016). In 60 Ugandan patients with human immunodeficiency virus (HIV) infections in whom pneumonia was treated with antimicrobial agents, patients with reduced airway bacterial diversity showed increased bacterial load and increased expression of matrix metalloproteinase (MMP)-9 and pro-inflammatory TNF-*α* (Iwai et al., 2014). In addition, the differences in the lower respiratory tract of patients with advanced HIV infection were much greater than those in healthy individuals. These studies suggest that the composition of the respiratory microbiota is correlated with immune response (Lozupone et al., 2013).

## 4 Respiratory microbiota and respiratory viral infections

**Figure 2:**
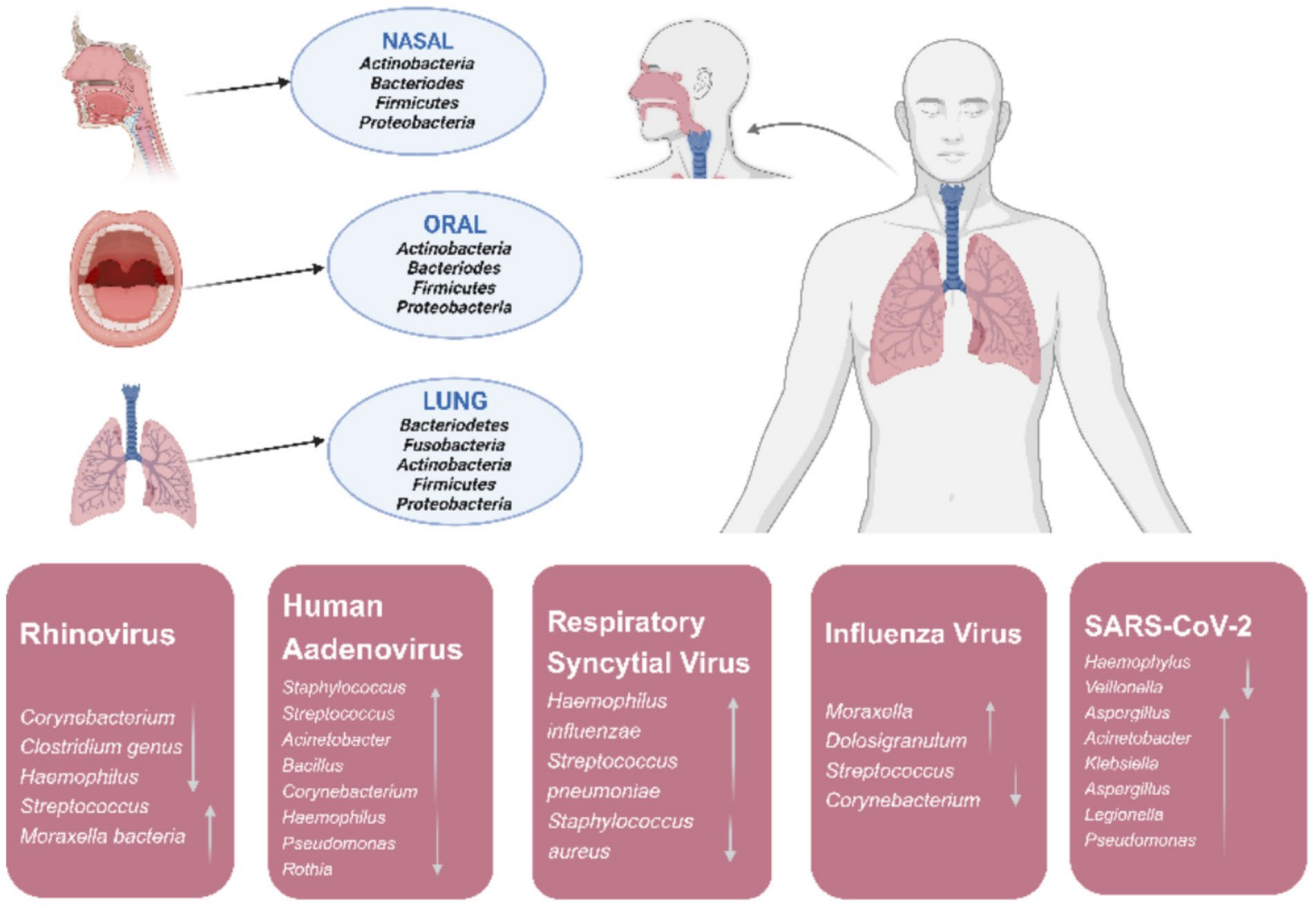
In a healthy state major phyla originating from the microbiota of nasal, oral, and lung in the human respiratory tract and changes of respiratory tract microbiota during respiratory viral infections.

The respiratory microbiota can interact with respiratory viruses at multiple levels. On the one hand, respiratory microbiota and its metabolites can promote the proliferation of respiratory viruses and enhance their infectivity. Patients with cystic fibrosis are often susceptible to respiratory syncytial virus infection following infection with pathogenic *Pseudomonas aeruginosa*, influenza virus, rhinoviruses, and adenovirus (Kiedrowski and Bomberger, 2018). On the other hand, respiratory viruses can also cause secondary bacterial infections by damaging the host mucosal barrier, affecting immune function, and causing changes in the abundance and diversity of respiratory microbiota (Mazel-Sanchez et al., 2019) (Figure 2).

The novel coronavirus disease has triggered a global pandemic that has infected hundreds of millions of people and killed millions of people, with profound consequences for economies, societies, and public health systems worldwide. The severity of the coronavirus disease 2019 (COVID-19) is strongly correlated with the respiratory microbiome. As the severity of COVID-19 increases, oropharyngeal *α*-diversity tends to decrease in patients (Hernandez-Teran et al., 2021; Rueca et al., 2021; Bradley et al., 2022). The composition and dynamics of oropharyngeal microbiota were significantly associated with COVID-19 mortality. In patients with severe COVID-19, the relative abundances of *Hemophilus* and *Neisseria* decreased, which is consistent with previous studies (de Castilhos et al., 2022; Liu et al., 2015). *Veillonella*, especially *Veillonella parvula*, may be a potential biomarker of COVID-19 (Ma et al., 2021). In addition, the higher the abundance of *Streptococcus* at admission, the better the prognosis (Ren et al., 2021). The relative abundance of opportunistic pathogens (including the aforementioned bacterial species) in the nasopharynx of patients with severe COVID-19 was lower than that in patients with mild cases (de Castilhos et al., 2022). Patients with severe COVID-19 are more likely to have reduced *α*-diversity and increased bacterial load in their lungs than healthy individuals and patients with mild COVID-19 (Merenstein et al., 2021). A metatranscriptomics and metagenomics analysis of bronchoalveolar lavage fluid and sputum samples from 116 COVID-19 patients investigated the relationship between microbial composition and disease severity (mild, severe, and critical). It is indicated that disease severity increased with the Chao index. However, *Streptococcus* and *Rothia* abundance both decreased with disease progression from mild to critical. Firmicutes was the dominant microbial taxon in the mild group, while Bacteroidetes abundance increased in the severe group. Finally, the critical group was characterized by higher Actinobacteria and Proteobacteria abundance (Wang et al., 2025). These results suggest that SARS CoV-2 infection disrupts both the oropharyngeal microbiome and the microbiome of the lower respiratory tract. This microbial imbalance potentially increases the risk of secondary pulmonary infections (Wang et al., 2025).

Approximately 250,000–500,000 people die from influenza annually, placing a huge burden on public health (Javanian et al., 2021). Changes in the respiratory microbiome may affect susceptibility to influenza (Tsang et al., 2020), which is related to age (Huffnagle et al., 2017). In a mouse model, after infection with influenza A virus (IAV), the threshold of bacterial invasion decreased, which was mainly caused by the change in bacterial composition in the respiratory tract, but had limited association with the change in overall bacterial abundance. Symbiotic bacteria such as *Streptococcus infantis* and *Streptococcus mitis* may play a key role in resisting the overgrowth of these pathogens (Li et al., 2023).

Ding et al. (2019) found differences in the composition of the oropharyngeal microbiota in acute respiratory infections caused by different IAV subtypes. In patients with influenza A, the main species were *Atopobium* and *Prevotella*, whereas in patients with influenza B, *Bergeyella* and *Prevotella* dominated (Li et al., 2023). In addition, vaccination was also found to correlate with microbiota composition in cohorts of influenza A and B, which could mean that influenza vaccination not only prevents influenza infection but may also play a role in controlling secondary bacterial infections (Ding et al., 2019).

Rhinovirus (RV) infection has also been associated with changes in respiratory microbiome (Nakagome et al., 2014; Lehtinen et al., 2018). With an increase in RV load, the abundance of *Corynebacterium* and *Dolosigranulum* decreased, while the abundance of Hemophilus increased. In addition, the abundance of *Streptococcus* and *Moraxella* increased and decreased, respectively, with changes in the RV replication level. The enrichment of *Corynebacterium* and *Guignococcus* may help individuals with infection maintain normal respiratory physiological functions during rhinovirus infection, thereby reducing or preventing respiratory symptoms (Kloepfer et al., 2017).

Respiratory syncytial virus (RSV) infection is closely associated with nasopharyngeal microbiota. The main manifestation includes an increase in *H. influenzae* and *S. pneumoniae*. Additionally, there appeared to be an interaction between RSV and these two bacteria (de Steenhuijsen Piters et al., 2016). Kanmani et al. (2017) found that the injection *Corynebacterium pseudodiphtheriticum*, one of the components of the upper respiratory tract microbiota in healthy people, into the nasal cavity of young mice enhanced their resistance to RSV and pneumococcal secondary infection.

Human adenovirus (HAdV) is an important infectious respiratory tract pathogen in children, accounting for 4–10% of community-acquired pediatric pneumonia cases (Jobran et al., 2018). Adenoviruses can cause an increase in *Staphylococcus, Streptococcus, Acinetobacter*, *Bacillus, and Corynebacterium*, and a decrease in *Hemophilus, Pseudomonas*, and *Rothia*. Adenoviral infection is often accompanied by mycoplasma co-infection (Zhong and Dong, 2021). Children with HAdV infection have higher microbial diversity in their lungs than patients with *Mycoplasma pneumoniae* infection. Compared with patients with *M. pneumoniae* infection alone, patients with HAdV infection had decreased BALF microbial richness and increased *β*-diversity (Zhou et al., 2022). The reason may be that *M. pneumoniae* inhibits the growth of other bacteria by directly competing with or activating bacterial clearance mechanisms, whereas HAdV infection damages lung epithelial cells and suppresses the immune response, thus promoting bacterial growth (Wang et al., 2019; Yang et al., 2004).

## 5 Respiratory microbiota and malignant tumors

The oral microbiota plays a major role in oral health and systemic diseases, including malignancies. A study on oral microbiota and lung cancer risk in low-income populations in the southeastern United States analyzed 156 cases of lung cancer (73 European Americans and 83 non-European Americans) and 156 individually matched controls in a cohort study using 16S rRNA gene sequencing to analyze oral microbiota in oral rinse samples and evaluate the association between individual bacterial abundance or prevalence and lung cancer risk. Oral microbiota may play a role in the development of lung cancer (Shi et al., 2021). Jin et al. (2019) analyzed the BALF of 91 patients with lung cancer, 29 patients with non-malignant lung disease, and 30 healthy subjects using metagenomics and found that compared to healthy subjects, the airway microbial abundance of patients with lung cancer was reduced, and the microbiome of patients with non-malignant lung disease was similar to that of patients with lung cancer. *Bradyrhizobium japonicum* was found only in patients with lung cancer, whereas *Acidovorax* was found in patients with lung cancer and non-malignant lung diseases. This study showed reduced microbiome abundance in patients with lung cancer and that microbiome specific biomarkers may help diagnose lung cancer in patients when lung biopsy is not feasible. Zhang et al. (2020) screened for colorectal cancer by detecting the oral microbiota and found that the microecological imbalance of oral pathogens, such as *Clostridium, Prevotella,* and *Porphyromonas* may be the main risk factor for colorectal cancer. The detection of oral microbial markers may serve as an effective, non-invasive method for colorectal cancer screening. Patients with pancreatic cancer have oral microbiota dysbiosis. DNA microarrays were used to compare the oral salivary microbiota of patients with pancreatic cancer, chronic pancreatitis, and healthy controls. The abundance of *Neisseria elongatus* and *S. mitis* decreased significantly in patients with pancreatic cancer. The abundance of *Granulicatella adiacens* was significantly higher in patients with chronic pancreatitis, and significantly lower than *S. mitis* (Vogtmann et al., 2020).

## 6 Administration of antibiotics and probiotics

The effects of antibiotics, the routine clinical treatment of respiratory infections, on the respiratory microbiota are not always favorable. For example, long-term use of macrolides can significantly alter the composition of the microbiota in the lower respiratory tract (Taylor et al., 2019; Ritchie and Singanayagam, 2020), and mice treated with neomycin were more likely to be susceptible to influenza than the control group (Ichinohe et al., 2011). In addition, the use of antibiotics can lead to selection pressure. One study compared the oropharyngeal microbial composition and related functional changes between healthy individuals and patients with moderate to severe COVID-19 (Wu et al., 2023). The results showed that disease-causing pathogens enriched in patients with COVID-19 had higher virulence and resistance, suggesting that antibiotic use may make the disease more difficult to control. Probiotics play an important role in microbial therapy as a common means of regulating microbiota. The use of gut-lung or gut-respiratory axis interactions to regulate the airway response by oral probiotics has become a mainstream strategy (Dang and Marsland, 2019). Oral microbiological therapy with lactic acid bacteria can help reduce the risk of respiratory failure, as shown in a small clinical study on patients COVID-19 (d'Ettorre et al., 2020). Furthermore, ClinicalTri-als.gov has registered several clinical trials investigating the effectiveness of probiotics (such as *Lactobacillus* or *Bifidobacterium*) in preventing or treating respiratory viral infections. These clinical trials not only reflect the recognition of the potential clinical value of probiotics by the scientific community but also indicate the significant clinical and economic benefits that may be brought about in the future.

Compared with the indirect effect of oral probiotics on respiratory health through the entero-lung axis, the direct application of microbiota to the respiratory tract to exert activity at the site of infection may be more effective in regulating the susceptibility to infection and its course. Previous studies demonstrated the effectiveness of local application. For example, a single prophylactic fogging treatment with *H. influenzae* lysate successfully prevented the occurrence of influenza pneumonia (Tuvim et al., 2009), whereas an intranasal injection of fermentative *Lactobacillus* CJL-112 L promoted the production of specific protective IgA and enhanced immune defense in a mouse model of influenza infection (Yeo et al., 2014).

*Lactobacillus* is considered a promising candidate strain owing to its various probiotic properties and potential for local application in the airway (De Boeck et al., 2021; De Boeck et al., 2020), Several studies have demonstrated the feasibility and safety of its airway administration. However, determining the optimal probiotic strains, mixtures, dosages, and formulations remains challenging in clinical practice.

In addition, the metabolites of the microbiota, as key signals for the microbiota-host interaction, also show considerable potential as a treatment for lung infections (Montassier et al., 2023). The discovery of bacteria derived host isoenzymes in the gut provides a new therapeutic approach for metabolic diseases (Wang et al., 2023), suggesting that similar mechanisms may also exist in the respiratory tract; however, their specific existence and mechanism of action need to be further explored.

## 7 Conclusion

In recent years, important progress has been made in the study of respiratory microbiota, and the main microbiota types in each anatomical region of the upper and lower respiratory tracts have been identified. Respiratory infections are often accompanied by a decline in microbial diversity, an increase in microbial load, and an increase in the abundance of opportunistic pathogens, especially in patients in intensive care unit (ICU), where the abundance of multiple bacteria is correlated with patient outcomes. Recent studies on respiratory microbiota in disease states have identified microbial signatures associated with differential diagnosis, disease severity, and prognosis. However, the lack of consistency among the results of different studies makes it challenging to clearly define respiratory microbiota dysbiosis.

Even within the course of a single disease, multiple forms of dysregulation may occur. The phenotypes of dysregulation of the respiratory microbiota are inconsistent across cohorts, infection types, and disease progression, making the discovery of common markers of the respiratory microbiota challenging. Future studies should shift from crosssectional designs and correlation studies to longitudinal sampling methods, consider geographical characteristics and experimental conditions, and establish animal models that can closely simulate the human respiratory microbiota to better control the microbiota and host conditions to verify the mechanism of microbial action.

At present, clear evidence for the local use of probiotics in the respiratory tract to regulate respiratory microbiota and prevent respiratory infections is lacking. Although intermittent drops of oral symbiotic bacteria in the lower respiratory tract altered the host susceptibility to respiratory pathogens in multiple animal experiments (Jin et al., 2019; Zhang et al., 2020), there is still a lack of institutional studies and feasible implementation plans in this field. Conducting safe and meaningful clinical trials at present is challenging.
